# Kinetics of Early Innate Immune Activation during HIV-1 Infection of Humanized Mice

**DOI:** 10.1128/JVI.02123-18

**Published:** 2019-05-15

**Authors:** Jessica Katy Skelton, Ana Maria Ortega-Prieto, Steve Kaye, Jose Manuel Jimenez-Guardeño, Jane Turner, Michael H. Malim, Greg J. Towers, Marcus Dorner

**Affiliations:** a Section of Virology, Department of Medicine, Imperial College London, London, United Kingdom; b Molecular Diagnostics Unit, Imperial College London, London, United Kingdom; c Department of Infectious Diseases, School of Immunology and Microbial Sciences, King’s College London, London, United Kingdom; d Division of Infection and Immunity, University College London, London, United Kingdom; Ulm University Medical Center

**Keywords:** human immunodeficiency virus, humanized mouse, innate immunity

## Abstract

Human immunodeficiency virus type 1 (HIV-1) infection is associated with aberrant immune activation; however, most model systems for HIV-1 have been used during established infection. Here, we utilize ultrasensitive HIV-1 quantification to delineate early events during the eclipse, burst, and chronic phases of HIV-1 infection in humanized mice. We show that very early in infection, HIV-1 suppresses peripheral type I interferon (IFN) and interferon-stimulated gene (ISG) responses, including the HIV-1 restriction factor IFI44. At the peak of innate immune activation, prior to CD4 T cell loss, HIV-1 infection differentially affects peripheral and lymphoid Toll-like receptor (TLR) expression profiles in T cells and macrophages. This results in a trend toward an altered activation of nuclear factor κB (NF-κB), TANK-binding kinase 1 (TBK1), and interferon regulatory factor 3 (IRF3). The subsequent type I and III IFN responses result in preferential induction of peripheral ISG responses. Following this initial innate immune activation, peripheral expression of the HIV-1 restriction factor SAM domain- and HD domain-containing protein 1 (SAMHD1) returns to levels below those observed in uninfected mice, suggesting that HIV-1 interferes with their basal expression. However, peripheral cells still retain their responsiveness to exogenous type I IFN, whereas splenic cells show a reduction in select ISGs in response to IFN. This demonstrates the highly dynamic nature of very early HIV-1 infection and suggests that blocks to the induction of HIV-1 restriction factors contribute to the establishment of viral persistence.

**IMPORTANCE** Human immunodeficiency virus type 1 (HIV-1) infection is restricted to humans and some nonhuman primates (e.g., chimpanzee and gorilla). Alternative model systems based on simian immunodeficiency virus (SIV) infection of macaques are available but do not recapitulate all aspects of HIV-1 infection and disease. Humanized mice, which contain a human immune system, can be used to study HIV-1, but only limited information on early events and immune responses is available to date. Here, we describe very early immune responses to HIV-1 and demonstrate a suppression of cell-intrinsic innate immunity. Furthermore, we show that HIV-1 infection interacts differently with innate immune responses in blood and lymphoid organs.

## Abstract (precis)

Human immunodeficiency virus type 1 (HIV-1) infection is restricted to humans and some nonhuman primates (e.g., chimpanzee and gorilla). Alternative model systems based on simian immunodeficiency virus (SIV) infection of macaques are available but do not recapitulate all aspects of HIV-1 infection and disease. Humanized mice, which contain a human immune system, can be used to study HIV-1, but only limited information on early events and immune responses is available to date. Here, we describe very early immune responses to HIV-1 and demonstrate a suppression of cell-intrinsic innate immunity. Furthermore, we show that HIV-1 infection interacts differently with innate immune responses in blood and lymphoid organs.

## INTRODUCTION

More than 37 million people are currently infected with human immunodeficiency virus (HIV) (1). Since HIV-1 stably integrates into the host cell genome of CD4 T cells, no curative therapies are currently available (2). The narrow species tropism of HIV-1 limits natural infection to humans, mainly due to incompatibility of host factors and the presence of restriction factors in nonhuman cells (3, 4). Even though surrogate model systems are available (e.g., simian immunodeficiency virus [SIV] [SIVmac]), these do not recapitulate all the complex host-pathogen interactions evolved by HIV-1, mostly because they present preadaptation strains (i.e., SIVcpz or SIVsm) or HIV-2 progenitors (5, 6). Additionally, SIV and HIV-2 encode an additional accessory protein, Vpx, which alters disease progression by promoting replication in myeloid cells by blocking the action of the restriction factor SAM domain- and HD domain-containing protein 1 (SAMHD1) (7, 8). Furthermore, *in vitro* models of HIV-1 persistence in T cells generally require preactivation of the cells, which results in their proliferation and altered phenotype. This is in stark contrast to infection *in vivo*, where any cellular activation is driven by endogenous processes (9). Even though more physiological model systems have been developed, including *ex vivo* tissue explant models (10, 11) or cytokine-driven models of HIV-1 latency (12, 13), they often rely on extensive manipulation of natural physiology, are often challenging to accurately control, and have not yet been demonstrated to be equivalent to naturally infected cells.

Humanized mice have been developed to bridge this gap, facilitating HIV-1 infection using human cells *in vivo* (14). These models largely rely on the ability of human hematopoietic stem cells (HSC) to utilize the murine bone marrow stem cell niche to reconstitute highly immunodeficient mice with all major human hematopoietic lineages (15). Even though advanced model systems incorporating implanted human fetal liver and thymus have been developed (16), the limited availability of human fetal tissue as well as ethical considerations make human HSC-engrafted humanized mice the most widely used and tractable model (15). Even though many humanized mouse models have been used to study HIV-1 infection, currently, no data are available on very early events in the path to HIV-1 persistence. In addition, early innate immune activation by HIV-1 through type I interferon (IFN) is still poorly understood, and even less is known in regard to the role of type III IFN in HIV-1 infection. Furthermore, it remains unclear to what extent HIV-1 infection activates (17–19) or interferes with (20–22) innate immune activation and IFN-based signaling. Even though many IFN-stimulated genes (ISGs) with antiretroviral activity have been described (3, 23), it remains unclear to what extent these are present, induced, repressed, or evaded during HIV-1 infection *in vivo*.

Here, we demonstrate that ultrasensitive detection can be used to distinguish very early eclipse and burst phases preceding the onset of CD4 T cell loss in humanized mice. These phases give clear insight into the differential innate immune activation stages involved in establishing HIV-1 persistence as well as illustrating how HIV-1 subverts host efforts to restrict infection. This offers valuable insight into how HIV-1 interacts with the innate immune system early during infection and explains why common IFN-stimulated HIV-1 restriction factors may be limited in their capacity to control infection.

## RESULTS

### HIV-1 infection exhibits a multiphasic kinetic, stabilizing after 3 weeks.

**FIG 1:**
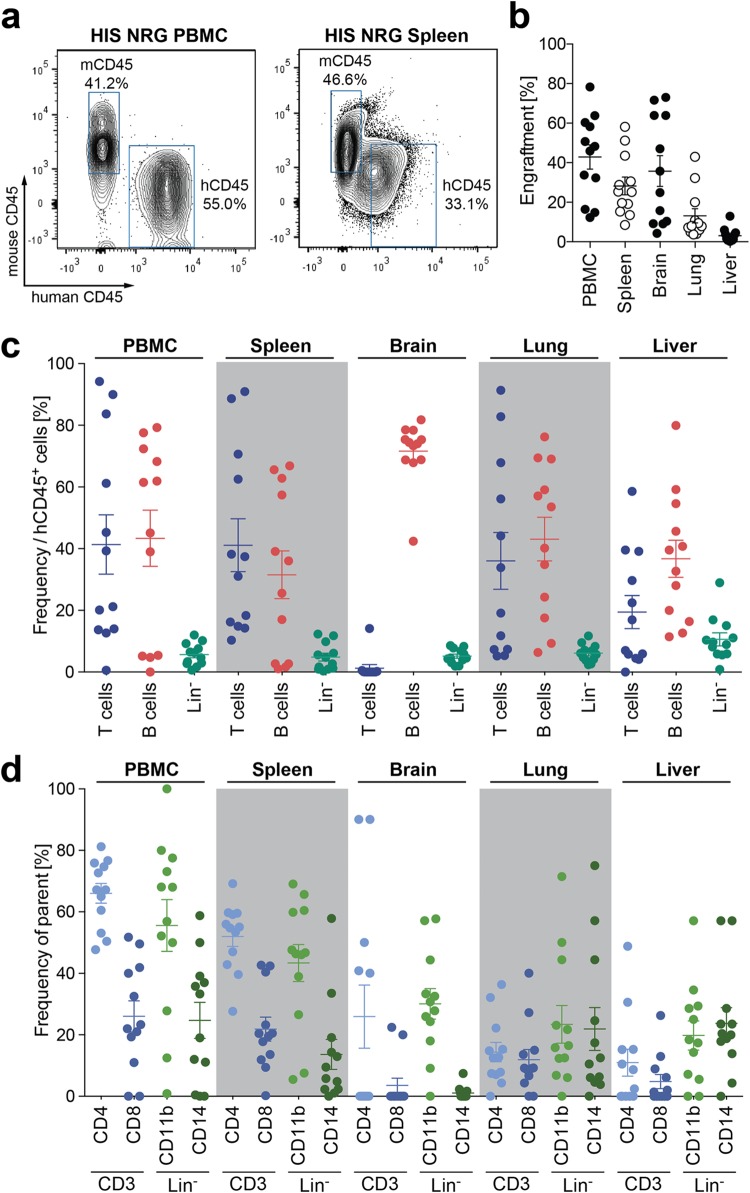
Humanized mice engrafted with human cells in all major organs. (a) Representative flow cytometry plots depicting human cell populations in humanized (HIS) NRG mice in both the peripheral blood and spleen. (b) Percentage of human cell engraftment in HIS mice in PBMC, spleen, brain, lung, and liver tissue. Engraftment is depicted as the percentage of human CD45^+^ cells in the total leukocyte population (e.g., human CD45 [hCD45] and mouse CD45 [mCD45]). (c) Frequency of CD3^+^ T cells, CD19^+^ B cells, and lineage-negative (Lin^−^) cells (CD3^−^ CD19^−^) in PBMC, spleen, brain, lung, and liver. (d) Frequencies of CD4^+^ and CD8^+^ T cells (within the CD3^+^ cell population) and of CD14^+^ monocytes and CD11b^+^ macrophages (within the lineage-negative population), as depicted in panel c. Data shown are from 12 humanized NRG mice reconstituted with cells from 3 different CD34 HSC donors.

Despite many studies utilizing humanized mice to model HIV-1 infection (15), little information was available on very early virological and immunological events following infection. The generation of humanized mice by intrahepatic injection of HSC into sublethally irradiated NOD.Cg-*Rag1^tm1Mom^IL2rg^tm1Wjl^*/SzJ (NRG) mice resulted in multilineage engraftment of human immune cells in all major organs within 3 months, generating HIS mice (Fig. 1a and b). This included CD3^+^ T cells, CD19^+^ B cells, as well as lineage-negative cells (Fig. 1c). CD3^+^ CD4^+^ T helper cells, the main target for HIV-1 infection, as well as CD3^+^ CD8^+^ cytotoxic T cells were present at physiological frequencies, and despite their lower repopulation, both CD11b^+^ macrophages and CD14^+^ monocytes were present in all major organs (Fig. 1d).

**FIG 2:**
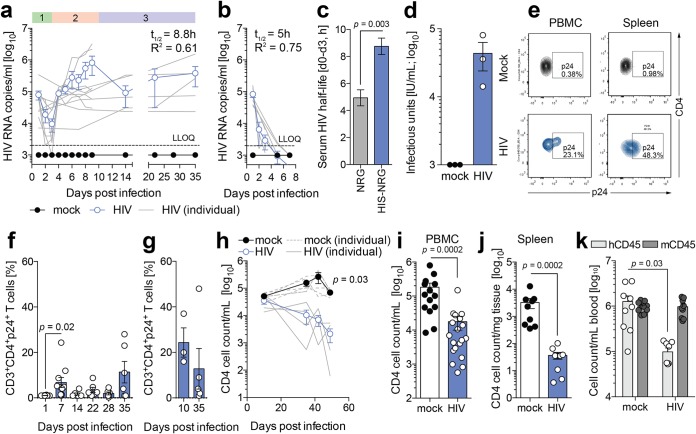
HIV-1 results in distinct phases of infection in humanized mice. (a) Serum HIV-1 RNA kinetic in humanized NRG mice following infection with 2.1 IU RT from NL4.3 (BaL Env), as measured by two-step qRT-PCR. Data shown are means ± standard errors of the means (SEM) (blue circles) for 12 mice per cohort as well as individual animals (gray lines). Numbers indicate eclipse (1), burst (2), and chronic (3) phases of infection. LLOQ, lower limit of quantification. (b) Serum HIV-1 RNA kinetic in nonhumanized NRG mice following infection with 2.1 IU RT from NL4.3 (BaL Env), as measured by two-step qRT-PCR. Data shown are means ± SEM (blue circles) for 7 mice per cohort as well as individual animals (gray lines). (c) Serum HIV RNA half-life (t_1/2_) calculated between days 1 and 3 of infection of HIS NRG or NRG mice by one-phase exponential-decay least-squares fitting. (d) HIV-1 infectious units in plasma isolated from HIV-1-infected humanized mice at day 22 postinfection, as measured by GHOST cell titration. (e) Representative flow cytometry plots of CD3^+^ CD4^+^ p24^+^ T cells in both HIV-1-infected and uninfected HIS mice in peripheral circulation and splenic tissue. (f and g) Percentages of HIV-1-infected CD3^+^ CD4^+^ p24^+^ cells observed in PBMC (f) and splenic tissue (g) at days 10 and 35 after HIV-1 infection, as determined by flow cytometry and analyzed by a Wilcoxon matched-pairs signed-rank test with Bonferroni corrections. (h) Longitudinal CD4 T cell loss in peripheral blood of HIV-1-infected HIS mice. Data shown are means ± SEM (blue circles) for 5 mice per cohort as well as individual animals (gray lines). Statistical significance was determined between mock-infected and HIV-1-infected humanized mice at day 49 postinfection using a Mann-Whitney test with Bonferroni corrections. (i and j) Absolute CD4 T cell loss in PBMC (i) and spleen (j) of HIS mice 35 days following infection with 2.1 IU RT from NL4.3 (BaL Env). Data shown are means ± SEM (blue circles) for 19 and 9 mice per cohort, respectively, analyzed by Mann-Whitney test using Bonferroni corrections. (k) Cell counts of human and mouse CD45 leukocytes in HIS mice before and 35 days after HIV-1 infection. Data shown are means ± SEM for 9 uninfected and 10 HIV-1-infected mice. Statistical significance was determined with a Wilcoxon matched-pairs signed-rank test with Bonferroni corrections. For all statistical tests, a *P* value of <0.05 was deemed significant.

Infection of these mice with the CCR5-tropic HIV-1 recombinant virus NL4.3 (BaL Env) resulted in stable viremia, as determined by ultrasensitive reverse transcriptase quantitative PCR (qRT-PCR) (Fig. 2a). Notably, this method allowed us to observe three phases during infection: an eclipse phase up to day 4 followed by a burst phase of viral replication before expansion of HIV-1 RNA copies in the plasma of mice 4 to 9 days before stable peak viremia was reached (Fig. 2a). Nonhumanized control cohorts were utilized to demonstrate rapid virological decay in the absence of human cells (Fig. 2b). Strikingly, comparing the initial decay of HIV-1 RNA in the sera of HIV-1-infected humanized and nonhumanized mice within the first 3 days of the eclipse phase, the serum HIV-1 RNA half-life was significantly prolonged in mice containing human immune cells (Fig. 2c). At day 22 postinfection, *de novo*-produced infectious HIV-1 particles were detectable in the serum of infected mice, as determined by titration on GHOST cells (Fig. 2d). p24-expressing HIV-1-infected CD3^+^ CD4^+^ T cells were detectable with a peak at 7 days postinfection before contracting to stable levels, detected in both peripheral blood and splenic tissue of these mice (Fig. 2e to g). This shows that HIV-1 infection in humanized mice can be assessed at very early time points postinfection and that HIV-1 viremia initially contracts prior to the establishment of stable serum viremia.

Among the hallmarks of HIV-1 infection is the resulting longitudinal loss of CD4 T cells (24). Critically, and similar to HIV-1 infection in humans, in this model, HIV-1 infection resulted in an overall reduction of total CD4 T cell counts in peripheral blood and spleen (Fig. 2h to j). This progressively worsened and, in some respects, therefore recapitulated progression to AIDS in human infection (Fig. 2h to j). As expected, the infection perturbs the total human leukocyte engraftment compared to uninfected mice, leaving the murine CD45-expressing leukocyte population unchanged (Fig. 2k). This demonstrates that the trajectories of HIV-1 infection in humanized mice bear similarities to those observed in humans.

### HIV-1 distinctly suppresses early innate immune activation.

**FIG 3:**
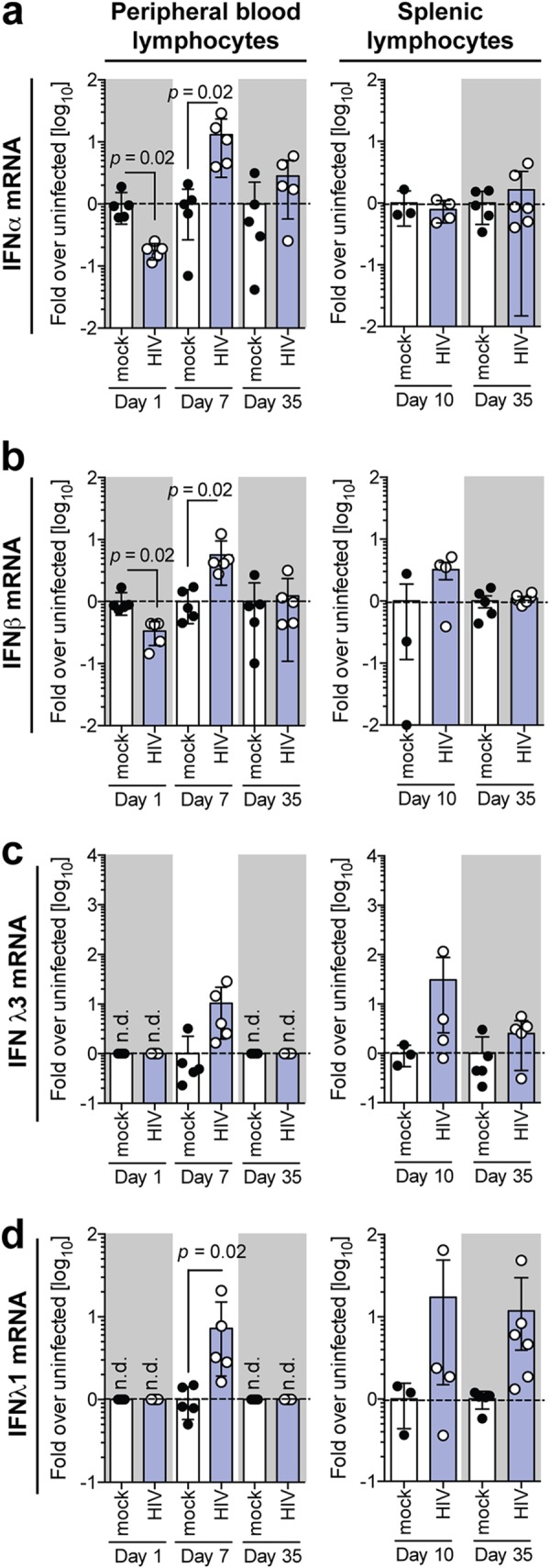
HIV-1 induces a compartmentalized interferon response in humanized mice. Shown are mRNA expression levels of IFN-α (a), IFN-β (b), IFN-λ3 (c), and IFN-λ1 (d) in peripheral blood lymphocytes and splenic lymphocytes isolated from HIV-1-infected humanized mice at days 1, 7, and 35 and at days 10 and 35, respectively. Statistical significance was determined between mock- and HIV-1-infected mice using a Mann-Whitney test with Bonferroni corrections. Data shown are means ± SEM for 5 mice per cohort for peripheral lymphocytes and 3 uninfected and 4 HIV-1-infected mice for splenic lymphocytes.

Reports of early HIV-1 infection in patients suggest a strong peripheral type I IFN response to infection (25). However, little information was available on the kinetics and the correlation of peripheral circulation with lymphoid responses. Thus, it remained unclear whether HIV-1 is able to suppress early host responses. Since HIV-1 infection is associated with immune hyperactivation (26), we aimed to measure the extent of innate immune activation throughout the establishment of HIV-1 persistence in humanized mice. To this end, we evaluated the induction of type I and III IFN at early, intermediate, and late time points. Interestingly, at only 24 h postinfection, HIV-1 specifically downregulated type I IFN (IFN-α and IFN-β) expression in peripheral blood lymphocytes (Fig. 3a and b), while neither of the type III IFNs was expressed (Fig. 3c and d). During the viral eclipse phase up to day 7, HIV-1 infection was associated with marked elevations of both type I and type III IFN in peripheral blood lymphocytes (Fig. 3a to d). Once HIV-1 infection was fully established at day 35 postinfection, type I and III IFN returned to baseline levels in the peripheral circulation (Fig. 3a to d).

**FIG 4:**
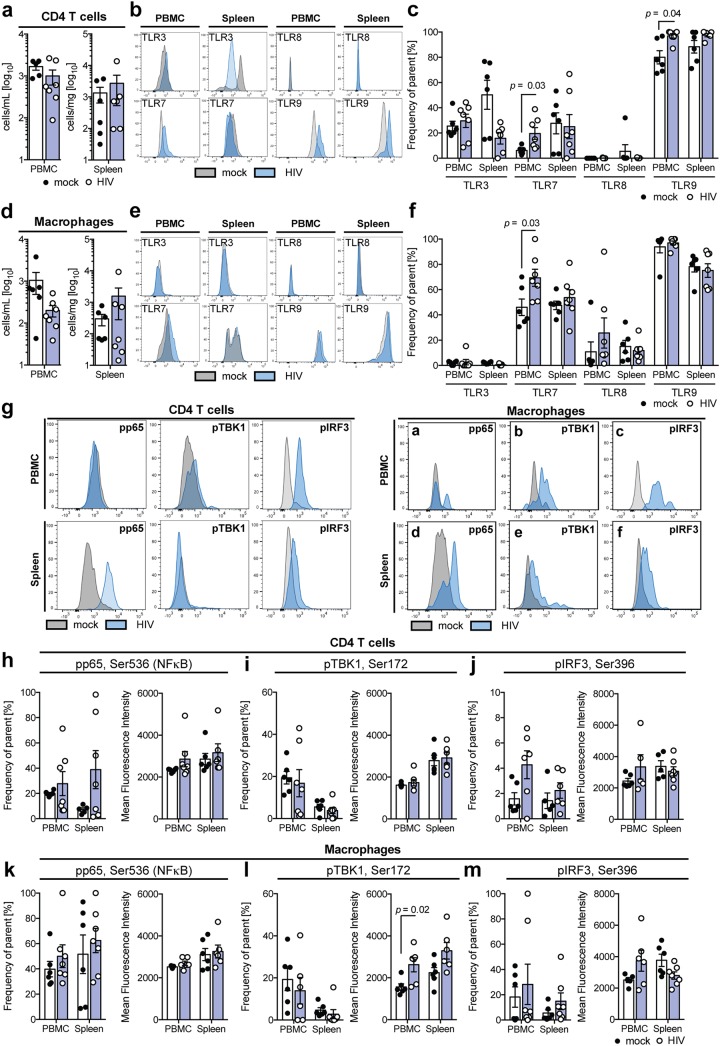
HIV-1 infection regulates TLR expression and induces differential pattern recognition in T cells and macrophages. (a) Total human CD4 T cell count in PBMC and spleen of uninfected and HIV-1-infected humanized mice 10 days following infection, as determined by flow cytometry. (b and c) Representative flow cytometry plots (b) and combined protein expression of TLR3, TLR7, TLR8, and TLR9 (c) in human CD4 T cells from PBMC and spleen of uninfected and HIV-1-infected human CD4 T cells 10 days following infection, as determined by flow cytometry and analyzed by a Mann-Whitney test with Bonferroni corrections. (d) Total human CD33^+^ macrophage counts in PBMC and spleen of uninfected and HIV-1-infected humanized mice 10 days following infection, as determined by flow cytometry. (e and f) Representative flow cytometry plots (e) and combined protein expression of TLR3, TLR7, TLR8, and TLR9 (f) in human CD33^+^ macrophages from PBMC and spleen of uninfected and HIV-1-infected human CD4 T cells 10 days following infection, as determined by flow cytometry and analyzed by a Mann-Whitney test with Bonferroni corrections. (g to m) Representative flow cytometry plots (g) and combined frequency and mean fluorescence intensity of protein phosphorylation of p65 Ser536 (NF-κB) (h and k), pTBK1 Ser172 (i and l), and pIRF3 Ser396 (j and m) in human CD4 T cells (h to j) and human CD33^+^ macrophages (k to m) in PBMC and spleen of uninfected and HIV-1-infected humanized mice 10 days following infection, as determined by flow cytometry and analyzed by a Mann-Whitney test with Bonferroni corrections. Data shown are means ± SEM for 6 uninfected and 7 HIV-1-infected mice. For all statistical tests, a *P* value of <0.05 was deemed significant.

To evaluate what mechanism drives the observed peripheral and splenic production of type I and III IFN at the peak of innate immune activation, prior to the HIV-1-associated depletion of CD4 T cells or macrophages (Fig. 4a and d), we evaluated the presence of pattern recognition receptors (PRR) able to sense viral RNA on peripheral as well as splenic CD4 T cells (Fig. 4b and c) and macrophages (Fig. 4e and f) 10 days following infection with HIV-1. At this point, HIV-1 infection resulted in a trend toward suppression of Toll-like receptor 3 (TLR3) protein expression on splenic but not peripheral CD4 T cells, whereas peripheral TLR7 and TLR9 protein expression was elevated after HIV-1 infection of both CD4 T cells and macrophages (Fig. 4b, c, e, and f).

To determine whether this differential TLR profile may be involved in the recognition of HIV-1 infection, we performed phosflow pathway analysis (39) of nuclear factor κB (NF-κB), TANK-binding kinase 1 (TBK1), and interferon regulatory factor 3 (IRF3) in peripheral and splenic CD4 T cells and macrophages 10 days following infection of humanized mice with HIV-1 (Fig. 4g to m). While splenic CD4 T cells respond to HIV-1 infection by inducing phosphorylation of the NF-κB subunit p65 at serine 536, this was not observed in peripheral CD4 T cells or macrophages (Fig. 4h and k). Even though many PRR pathways involve the phosphorylation of TBK1, only macrophages, but not CD4 T cells, exhibited elevated mean fluorescence intensities of phosphorylated TBK1 (Ser172) (Fig. 4i and l). Peripheral but not splenic CD4 T cells additionally showed a trend toward elevated levels of phosphorylated IRF3 (Ser396) (Fig. 4j and m).

Taken together, these results indicate that the HIV-1-induced induction of TLR7 and TLR9 expression in PBMC may be at least partially responsible for peripheral interferon responses.

### HIV-1-associated early immune activation is driven by both T cells and macrophages in peripheral but not lymphoid tissue.

**FIG 5:**
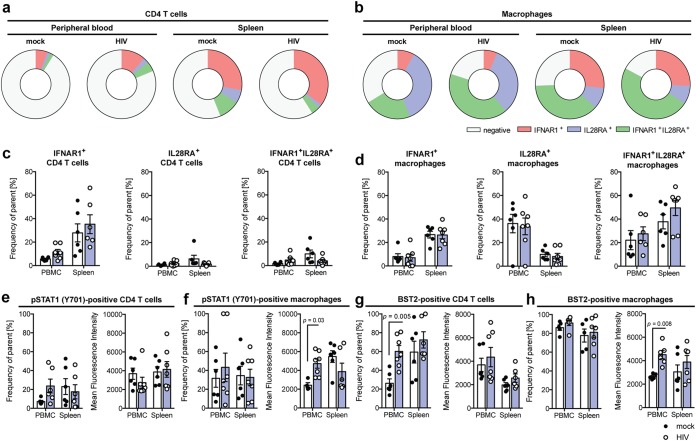
HIV-1 infection induces JAK/STAT signaling in human T cells and macrophages in humanized mice. (a and b) Mono- and coexpression of IFNAR1 and IL28RA on human CD4 T cells (a) and human CD33 macrophages (b) in PBMC and spleen of uninfected and HIV-infected humanized mice 10 days following infection. (c and d) Expression of IFNAR1 and/or IL28RA on CD4 T cells (c) and macrophages (d) in PBMC and spleen of uninfected and HIV-1-infected humanized mice 10 days following infection, as determined by flow cytometry. (e and f) Frequency and mean fluorescence intensity of STAT1 phosphorylated at tyrosine 701 in human CD4 T cells (e) and macrophages (f) in PBMC and spleen of uninfected and HIV-infected humanized mice 10 days following infection, as determined by flow cytometry and analyzed by a Mann-Whitney test with Bonferroni corrections. (g and h) Cell surface expression of BST2/tetherin on human CD4 T cells (g) and macrophages (h) 10 days following infection, as determined by flow cytometry and analyzed by a Mann-Whitney test with Bonferroni corrections. Data shown are means ± SEM for 6 uninfected and 7 HIV-1-infected mice. For all statistical tests, a *P* value of <0.05 was deemed significant.

Since HIV-1 infection resulted in a very compartmentalized, early IFN response in PBMC but not splenic tissue, we assessed the distribution of the respective receptors for type I and III IFN 10 days following HIV-1 infection by flow cytometry (Fig. 5a to d). As expected, the majority of macrophages in either PBMC or spleen expressed either IFNAR1, IL28RA, or IFNAR1 and IL28RA (Fig. 5b and d), whereas only a minority of CD4 T cells expressed predominantly IFNAR1 but not IL28RA (Fig. 5a and c).

To assess which cell populations responded to the production of type I and III IFN, we next examined the level of STAT1 phosphorylation at tyrosine 701, which is indicative of activated JAK/STAT signaling downstream of type I and III IFN receptors (Fig. 5e and f). This showed that macrophages in PBMC but not in spleen exhibited activated JAK/STAT signaling in response to HIV-1 infection, indicative of the observed production levels of type I and III IFN (Fig. 5e and f). This also directly correlated with the production of ISGs, as exemplified by tetherin/BST2, which was preferentially induced by HIV-1 infection in CD4 T cells and macrophages in peripheral circulation but was not induced in splenic CD4 T cells or macrophages.

**FIG 6:**
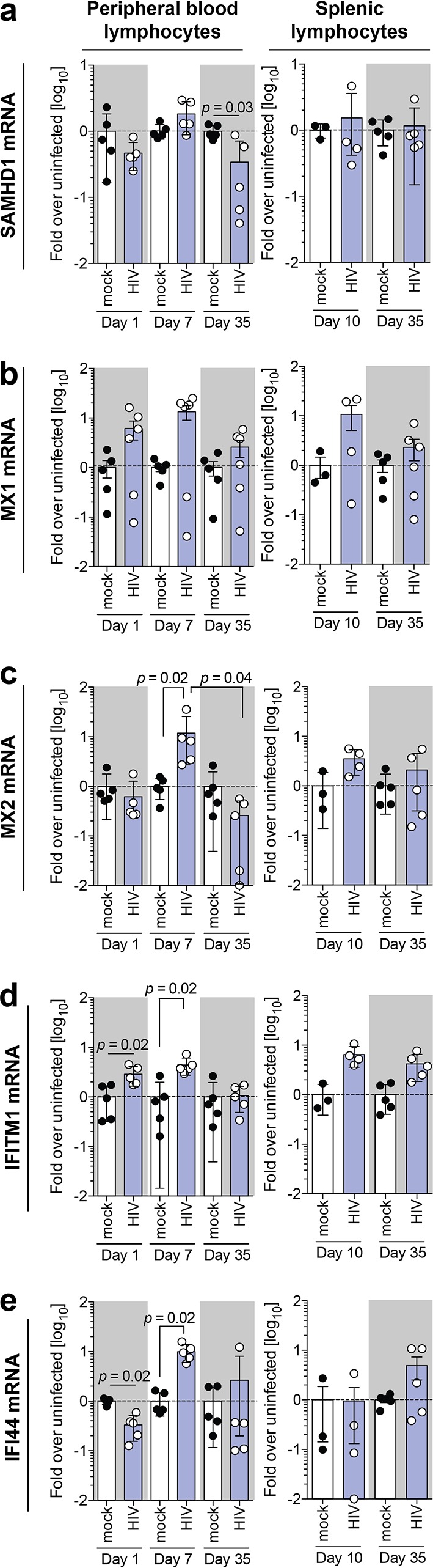
Dynamic gene expression profiles of key HIV-1 restriction factors indicate that early expression is perturbed. Shown are mRNA expression levels of SAMHD1 (a), MX1 (b), MX2 (c), IFITM1 (d), and IFI44 (e) in peripheral blood lymphocytes and splenic lymphocytes isolated from HIV-1-infected humanized mice at days 1, 7, and 35  and at days 10 and 35, respectively. Data shown are means ± SEM for 5 mice per cohort for peripheral lymphocytes and 3 uninfected and 4 HIV-1-infected mice for splenic lymphocytes. Statistical significance was determined using a Mann-Whitney test for unpaired mock- and HIV-1-infected mice and a Wilcoxon matched-pairs signed-rank test for paired HIV-1-infected mice between days 1, 7, and 35, both with Bonferroni corrections. For all statistical tests, a *P* value of <0.05 was deemed significant.

This compartmentalized IFN response additionally translated to the selective induction of well-known ISGs and HIV-1 restriction factors, including myxovirus resistance protein 2 (MX2), IFITM1, and IFI44, in PBMC at the peak of the initial HIV-1 replication burst (Fig. 6a to e). Surprisingly, however, both at the eclipse phase of infection as well as during late-stage chronic infection, HIV-1 infection suppressed baseline levels of IFI44 and showed similar trends with SAMHD1 and MX2 expression, indicating that HIV-1 actively shapes its environment.

Taken together, these results demonstrate that during early establishment of HIV-1 persistence in this model, innate immune activation in different compartments is highly dynamic, exhibiting signs of HIV-1-associated innate immune evasion and repression of expression of key HIV-1 restriction factors.

### Exogenous type I IFN can override HIV-1-induced repression of restriction factors only in peripheral circulation.

**FIG 7:**
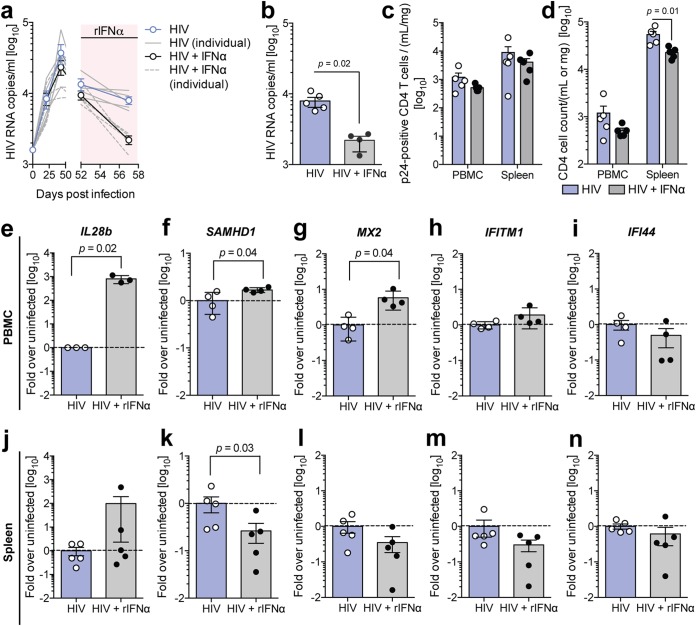
HIV-infected peripheral but not splenic cells retain their responsiveness to exogenous interferon. (a) Longitudinal serum HIV-1 RNA copies in humanized mice upon treatment with 1,000 IU/g rIFN-α intraperitoneally for 5 consecutive days (red shading). (b) Serum HIV RNA copies in untreated and rIFN-α-treated mice 5 days after treatment, as determined by HIV qRT-PCR and analyzed by a Mann-Whitney test with Bonferroni corrections. (c) Number of p24-positive CD4 T cells in PBMC and spleen of untreated and rIFN-α-treated mice 5 days after treatment, as determined by flow cytometry. (d) CD4 T cell counts in PBMC and spleen of untreated and rIFN-α-treated mice 5 days after treatment, as determined by flow cytometry and analyzed by a Mann-Whitney test with Bonferroni corrections. (e to n) mRNA expression of IL28b (e and J), SAMHD1 (f and k), MX2 (g and l), IFITM1 (h and m), and IFI44 (i and n) in PBMC (e to i) and spleen (j to n) of untreated and rIFN-α-treated mice 5 days after treatment, as determined by qRT-PCR and analyzed by a Mann-Whitney test with Bonferroni corrections. Data shown are means ± SEM for 5 mice per cohort, and for all statistical tests, a *P* value of <0.05 was deemed significant.

Based on recent experimental studies of SIV infection of macaques, it is possible that type I IFN accelerates HIV-1 disease progression (27, 28). However, details on the responsiveness of HIV-1-infected cells to IFN and the subsequent expression of IFN-induced restriction factors are still lacking. To evaluate whether chronic HIV-1 infection durably suppresses the induction of IFN responses and HIV-1 restriction factors, we treated HIV-1-infected humanized mice with 1,000 IU/g/day exogenous recombinant IFN-α (rIFN-α) for five consecutive days. This treatment resulted in a significant reduction of circulating serum HIV-1 RNA levels (Fig. 7a and b), indicating that the elicited inflammatory responses partially inhibited HIV-1 replication. Notably, administration of rIFN-α in HIV-1-infected humanized mice exacerbated the observed HIV-1-associated CD4 T cell depletion, indicating that ISGs and proinflammatory cytokines induced by type I IFN contribute to HIV-1-associated pathogenesis (Fig. 7d).

As expected, neither IFN-α, IFN-β, nor IFN-λ1 mRNA expression was elevated by rIFN-α (data not shown), whereas IFN-λ3, which is itself IFN stimulated, was induced by 3 log_10_ units at the end of treatment (Fig. 7e and j). rIFN-α treatment resulted in statistically significant increases of both SAMHD1 and MX2 in PBMC of HIV-1-infected humanized mice, whereas IFITM1 and IFI44 remained unchanged (Fig. 7f to i). Notably, despite a >2-log_10_ induction of IFN-λ3 in response to rIFN-α treatment in splenocytes of HIV-1-infected humanized mice, HIV-1 restriction factors, including SAMHD1, MX2, IFITM1, and IFI44, remained unchanged or were downregulated by rIFN-α (Fig. 7k to n). This indicates that HIV-1 infection does not result in suppression of IFN responsiveness in peripheral circulation but induces a compartmentalized interference with the induction of HIV-1 restriction factors in lymphoid tissue.

## DISCUSSION

Chronic HIV-1 infection is associated with immune hyperactivation. Even though the kinetic of natural HIV-1 infection has been extensively studied in the past, limited information is available on very early events following infection. Even though surrogate models for HIV-1 infection have been used in the past to dissect this, vast genetic differences as well as the presence of the SIV accessory protein Vpx make it difficult to directly correlate data from nonhuman primates with HIV-1 infection in humans. The main challenges in utilizing human samples to study early events in HIV-1 infection are that they are nearly always limited to peripheral blood, making it impossible to translate findings to events within lymphatic systems, as well as logistical challenges associated with specimen collection during the initial phases of HIV-1 infection.

Utilizing humanized mice, we show that HIV-1 infection and subsequent persistence are established by as early as 4 days postinfection, as evidenced by comparing HIV-1 serum RNA loads to those of mice without humanization. This far precedes the significant loss of CD4 T cells, which becomes notable only after 1 month of infection.

Strikingly, HIV-1 is able to suppress baseline peripheral IFN and ISG levels very early following infection, before HIV-1 RNA levels become detectable in peripheral circulation. This early suppression may contribute to the spread of HIV-1-infected cells, since key HIV-1 restriction factors, such as SAMHD1 and MX2, are present in host cells at lower levels. Only 1 week following infection, when serum HIV-1 RNA is already detectable, there is a significant peripheral type I and III IFN signature. However, the lymphatic system, as evidenced by measurements in the spleen, does not experience type I IFN production and instead exhibits a type III IFN signature. This absence of splenic type I IFN responses coincides with a trend toward reduced expression of the RNA sensor TLR3 on splenic CD4 T cells. Additionally, in contrast to peripheral CD4 T cells, HIV-1 infection does not induce the expression of TLR7 in splenic CD4 T cells or macrophages. This suggests a skewing of HIV-1-induced signaling toward proinflammatory NF-κB, rather than IFN-inducing IRF3 signaling in T cells and macrophages in the spleen. This could contribute to the pyroptosis-induced CD4 T cell loss characteristic during HIV-1 infection (29). In contrast, peripheral CD4 T cells show a trend toward increased phosphorylation and activation of IRF3. This may, at least in part, explain the observed differential and compartmentalized type I and III IFN production.

This is furthermore underpinned by the absence of an ISG response in the spleen, whereas multiple ISG and HIV-1 restriction factors are upregulated on the mRNA and protein levels in the peripheral circulation. This absence of early IFN responses explains why HIV-1 exhibits an early reservoir expansion in the near absence of restriction by host factors. Notably, even after the early peripheral induction of IFN and ISG responses, persistence of HIV-1 is associated with reduced expression of select ISGs and restriction factors in peripheral blood, which further underpins the ability of HIV-1 to evade host restriction. This suppression of key HIV-1 restriction factors may render the host more susceptible to systemic dissemination of HIV-1 infection.

Despite this evasion mechanism, PBMC retain their responsiveness to type I IFN, as exogenous treatment with recombinant IFN-α results in the induction of SAMHD1, IL28b, and MX2. However, splenic cells of HIV-1-infected humanized mice, upon treatment with recombinant IFN-α, display an inverse response to type I IFN, resulting in repressed restriction factor expression. In particular, SAMHD1 is significantly downregulated after IFN treatment, suggesting that HIV-1 may have evolved means to facilitate its dissemination in lymphatic tissue despite immune activation. This cannot be explained by a potential hurdle for rIFN-α to penetrate to the spleen upon exogenous administration, since IL28b, which itself is IFN stimulated, is readily detectable in the spleen. Surprisingly, another study using BLT mice, which exhibit a more functional adaptive immune system with the T cells’ ability to mount protective antigen-specific immune responses, demonstrated that CD4 T cell loss is delayed and that MX1 levels are chronically elevated (30). We observe very similar elevations of MX1; however, MX1, in contrast to MX2, has not been shown to restrict HIV-1 infection, and a comparative analysis in humanized mice has not been previously performed (31). Thus, the different kinetics of CD4 T cell loss may be due to antigen-specific T cell responses against HIV-1 infection releasing IFN-γ. Additionally, another study demonstrated that blocking type I IFN signaling results in T cell recovery during HIV-1 infection, supporting the notion that type I IFN might be associated with disease progression (32).

Taken together, our data show that IFN and ISG responses during acute HIV-1 infection in humanized mice are highly dynamic, exhibiting evidence for early viral suppression and peak recognition of HIV-1 by the innate immune system during the initial burst of HIV-1 replication. The trend observed indicating reduced TLR3 expression on splenic CD4 T cells upon HIV-1 infection could additionally contribute to the lack of HIV-1 RNA recognition. Since most of the initially induced HIV-1 restriction factors are returning to baseline or below-baseline expression levels during chronic HIV-1 infection, this could explain why HIV-1 is largely uncontrolled at later stages during infection. The induction of other ISGs without direct antiretroviral action could potentially result from other activation pathways and does not necessarily reflect the responses of ISGs in general. Indeed, CXCL10, which is commonly used as a marker for IFN stimulation and was shown to be elevated in untreated HIV-1-infected patients (33), was recently shown to be induced via TLR7/9 activation (34) rather than through JAK/STAT signaling. Most strikingly, the observed very early suppression of IFN and ISG responses *in vivo* has not been observed thus far. This early interference of pathogen sensing and IFN activation by HIV-1 may function as a viral strategy to establish an early reservoir and to eliminate restriction factor roadblocks preventing the establishment of HIV-1 persistence. The mechanism by which HIV-1 infection results in the transcriptional downregulation or destabilization of IFN mRNA remains elusive. However, many host proteins as well as viral accessory proteins (e.g., Vpr) are directly packaged within the HIV-1 virion and could contribute to this early downmodulation of the IFN system. This suggests that careful future analysis of single-cell transcriptional responses *in vitro* and *in vivo* is required to delineate which restriction factors are present and functional in HIV-1-infected as well as -uninfected bystander cells.

## MATERIALS AND METHODS

### Generation of humanized mice.

NOD.Cg-*Rag1^tm1Mom^IL2rg^tm1Wjl^*/SzJ (NRG) mice were obtained from The Jackson Laboratory and housed and bred at the Imperial College London CBS animal facility. Human CD34^+^ hematopoietic stem cells (HSC) were isolated from human fetal livers as previously described (35). Less-than-4-day-old NRG mice were sublethally irradiated with 100 cGy and 4 h later were intrahepatically injected with 1 × 10^5^ purified CD34^+^ HSC. Each experimental cohort was designed with groups comprising equal engraftment levels and numbers of animals of each gender and more than one HSC donor (Table 1).

**TABLE 1:** Humanized mouse cohort information

Type of infection	No. of mice	No. of male/no. of female animals	No. of HSC donors
Mock	41	18/23	10
HIV	55	21/34	12

Total	96	96	12

### HIV-1 infection of humanized mice and interferon treatment.

Wild-type NL4.3 BaL was generated via transfection in 293T cells and then purified on a 20% sucrose cushion prior to injection, as previously described (36). Humanized mouse cohorts were generated based on equal numbers from different HSC donors, engraftment levels, age, and sex prior to infection. Humanized mice were intravenously injected with 2.1 IU of reverse transcriptase as measured by SG-PERT, as previously described (37), and mock-infected mice were subsequently injected with an equal volume of the vehicle. For the IFN-α-treated cohorts, HIV-1-infected humanized mice, which displayed stable viremia, were intraperitoneally injected with 1,000 IU/g of body weight IFN-α2 (Invitrogen, Paisley, UK) daily for 5 days, and blood and tissues were then immediately harvested.

### Isolation of leukocytes from murine tissues.

Murine peripheral blood was isolated longitudinally using tail vein bleeding. Peripheral blood mononuclear cells were isolated from whole blood using murine red blood cell lysis buffer (Alfa Aesar, MA, USA) according to the manufacturer’s protocol. Fresh tissue isolated from humanized mice was washed in phosphate-buffered saline (PBS) and digested using 0.1% (wt/vol) collagenase digestion buffer for 30 min at 37°C. The digested tissue was then homogenized through a 70-μm cell strainer and loaded onto a Ficoll gradient according to the manufacturer’s protocol, and the leukocyte “buffy” layer was washed in PBS and stained for flow cytometry.

### Isolation of RNA from peripheral blood mononuclear cells and splenic tissue.

Fresh splenic tissue was harvested from humanized mice, directly stored in RNAlater stabilization solution (Life Technologies, Carlsbad, CA, USA), and frozen at −80°C. Upon RNA extraction, the samples were thawed and resuspended in the appropriate amount of RLT buffer containing β-mercaptoethanol according to the weight of the tissue or cell number. The tissue was then processed using the TissueLyser LT instrument (Qiagen, Manchester, UK) for 5 min at maximum speed. The RNA was processed using QIAshredder columns (Qiagen), followed by direct RNA isolation using the RNeasy minikit (Qiagen), according to the manufacturer’s protocol.

### Flow cytometry analysis.

To evaluate engraftment, 50 μl of whole blood was collected from a superficial tail vein 12 weeks following intrahepatic injection, and PBMC were extracted as described above. The isolated cells were then stained using anti-mouse CD45-BV605 (BD Bioscience [BD], NJ, USA) and anti-human CD45-allophycocyanin (APC) (BD), and engraftment was measured by the number of human CD45^+^ cells in the total leukocyte population.

Evaluations of human cell populations during both reconstitution analysis in organs and HIV-1 infection studies were performed as described above, and staining also included anti-human CD3-peridinin chlorophyll protein (PerCP)-Cy5.5 (BD), anti-human CD4-APC-H7 (BD), anti-human CD8-Alexa Fluor 700 (AF700) (BD), anti-human CD14-BV510 (BD), anti-human CD19-phycoerythrin (PE) (BD), anti-human CD11b-BV421 (BD), anti-human IFNAR1-PerCP (Novus, Manchester, UK), anti-human IL28RA-PE (BioLegend, CA, USA), anti-human BST2 (BioLegend), and the Live/Dead fixable dead cell staining kit (Life Technologies). Additionally, intracellular staining was performed using the BD Cytofix/Cytoperm solution kit (BD) according to the manufacturer’s protocol, using the following antibodies: KC57-p24 (Beckman Coulter, CA, USA), anti-human TLR3-APC (Miltenyi, Bergisch Gladbach, Germany), anti-human TLR7-Alexa Fluor 405 (R&D Systems, MN, USA), anti-human TLR8-Alexa Fluor 350 (R&D Systems), and anti-human TLR9-fluorescein isothiocyanate (FITC) (Abcam, Cambridge, UK). For absolute cell counts, CountBright absolute counting beads for flow cytometry (Thermo Fisher, MA, USA) were added to each sample according to the manufacturer’s protocol. All data were analyzed using FlowJo software.

### Phosflow analysis of protein phosphorylation.

Cells isolated from humanized mice were prepared for flow cytometry as described above and stained with the following extracellular markers: anti-mouse CD45-BV605 (BD), anti-human CD45-APC (BD), anti-human CD3-APC-eFluor780 (Thermo Fisher), anti-human CD8-AF700 (BD), and anti-human CD33-BV711 (BD). Cells were fixed with Cytofix buffer (BD) at 37°C for 10 min. Following this, cells were permeabilized with permeabilization buffer III (BD) for 30 min at 4°C and stained with the following phosflow antibodies: anti-human pSTAT1-BV421 (BD), anti-human pp65-PerCP-eFluor710 (Life Technologies), anti-human pIRF3 S396-PE (Cell Signaling, MA, USA), and anti-human pTBK-1 (Cell Signaling).

### HIV-1 ultrasensitive RNA quantification.

Total RNA was isolated from <50 μl murine plasma-EDTA using the QIAamp viral RNA isolation kit (Qiagen) according to the manufacturer’s protocol. In the first round, extracts were amplified using seminested RT-PCR with a limited cycle number using the Superscript III one-step RT-PCR system (Invitrogen) with primers (SID1 [5′-AAGACAGCAGTACAAATGGCAGT-3′] and SID2 [5′-TACTGCCCCTTCACCTTTCCA-3′]) targeting the HIV-1 integrase genomic region. The internal control RNA is a transcript of the integrase gene with the probe-binding region containing a randomized sequence of 25 nucleotides. The second round used the subsequent DNA product from the seminested RT-PCR as the template for quantitative PCR (qPCR), and HIV copies were detected using the QuantiTect probe PCR kit (Qiagen), using the following primers and probes: primers SID2 and SID3 (5′-CAATTTTAAAAGAAAAGGGGGGATT-3′), the HIV-1 probe 5′-FAM (6-carboxyfluorescein)–CGGGTTTATTACAGGGACAGCAGA–TAMRA (6-carboxytetramethylrhodamine)-3′, and the internal control probe 5′-VIC–CTGGGTAGAGTAGTCACAGAATGCG–BHQ (black hole quencher)-3′.

### Gene expression analysis.

Isolated RNA extracted from cells or tissue was converted into cDNA using a high-capacity cDNA reverse transcription kit (Applied Biosystems, Carlsbad, CA, USA) according to the manufacturer’s protocol. Quantification of human IFN-α, IFN-β, IFN-λ3, and IFN-λ1 and ISG expression of SAMHD1 (5′-TCACAGGCGCATTACTGCC-3′ and 5′-GGATTTGAACCAATCGCTGGA-3′), myxovirus resistance protein 1 (MX1), MX2 (5′-CAGCCACCACCAGGAAACA-3′ and 5′-TTCTGCTCGTACTGGCTGTACAG-3′), IFITM1, and IFI44 were evaluated using SYBR green PCR master mix (Life Technologies) on the ViiA 7 real-time PCR system instrument (Applied Biosystems) using previously described primer sequences (38). Subsequent mRNA levels were normalized to the glyceraldehyde-3-phosphate dehydrogenase (GAPDH) (5′-AGGGCTGCTTTTAACTCTGGT-3′ and 5′-CCCCACTTGATTTTGGAGGGA-3′) expression level. All primers were generated by Life Technologies.

### Statistical analysis.

All statistical analyses were performed using GraphPad Prism v6.0, and data were evaluated for statistical significance between experimental cohorts and conditions using one-phase exponential decay with a least-squares test, ordinary one-way analysis of variance (ANOVA) with Bonferroni’s multiple-comparison test. For the comparison of mock-infected with HIV-1-infected animals, the median-based unpaired Mann-Whitney test was performed with Bonferroni corrections. In contrast, a comparison of the same HIV-1-infected humanized mice at different time points was performed using the median-based paired Wilcoxon matched-pairs signed-rank test with Bonferroni corrections. For each statistical analysis, a *P* value of <0.05 was considered significant.

### Safety/biosecurity.

All work with infectious agents was conducted in biosafety level 3 facilities, approved by the Health and Safety Executive of the United Kingdom and in accordance with local rules, at Imperial College London, United Kingdom.

### Statement on animal ethics.

All work was approved by the local genetic manipulation (GM) safety committee of Imperial College London, St. Mary’s Campus (center number GM77), and the Health and Safety Executive of the United Kingdom and carried out in accordance with approved guidelines. All animal research described in this study was approved and carried out under a United Kingdom Home Office license, PPL 70/8219, in accordance with the approved guidelines under the Animals (Scientific Procedures) Act 1986 (ASPA).
